# Vector-Borne and Zoonotic Pathogens in Raccoon Dogs (*Nyctereutes procyonoides*) and Raccoons (*Procyon lotor*) from Schleswig-Holstein, Germany

**DOI:** 10.3390/pathogens13030270

**Published:** 2024-03-21

**Authors:** Jana C. Klink, Alexandra Rieger, Peter Wohlsein, Ursula Siebert, Anna Obiegala

**Affiliations:** 1 Institute for Terrestrial and Aquatic Wildlife Research, University of Veterinary Medicine Hannover, Foundation, 30559 Hannover, Germany; jana.christina.klink@tiho-hannover.de (J.C.K.); alexandra.rieger@tiho-hannover.de (A.R.); 2 Department of Pathology, University of Veterinary Medicine Hannover, Foundation, 30559 Hannover, Germany; peter.wohlsein@tiho-hannover.de; 3 Institute of Animal Hygiene and Veterinary Public Health, University of Leipzig, 04103 Leipzig, Germany; anna.obiegala@vetmed.uni-leipzig.de

**Keywords:** tick-borne diseases, vector-borne diseases, emerging infectious diseases, raccoon dog, raccoon, invasive species, *Leptospira*, *Rickettsia*, *Borrelia*, *Borreliella*

## Abstract

Raccoon dogs (*Nyctereutes procyonoides*) and raccoons (*Procyon lotor*) are invasive alien species originating from East Asia and North America, respectively. They are discussed as vectors and reservoirs for various infectious diseases, including vector-borne and zoonotic pathogens, and are therefore a potential threat to human and domestic animal health, as well as to biodiversity and conservation. In the years 2021 and 2022, 110 raccoon dogs (*Nyctereutes procyonoides*) and 30 raccoons (*Procyon lotor*) were screened via qPCR for the presence of *Leptospira* spp., *Rickettsia* spp. and *Borreliella* spp. in the German federal state of Schleswig-Holstein as part of a health and risk assessment study. *Borreliella* spp. were confirmed in one raccoon dog and one raccoon, identified as *Borreliella afzelii* in the raccoon. *Leptospira* spp. were found in 21 (19.44%) raccoon dogs and 2 (6.90%) raccoons. In five raccoon dogs, *Leptospira* spp. were identified as *Leptospira borgpetersenii*, *Leptospira kirschneri* and *Leptospira interrogans.*

## 1. Introduction

Zoonotic pathogens originating in wildlife have gained attention worldwide, with numerous wildlife species acting as reservoirs for pathogens that are a risk for human and domestic animal health, but also a threat to biodiversity and conservation [1,2]. Increased contact of humans and domestic animals with wildlife due to population growth, urbanisation and habitat encroachment leads to a growing risk of disease spread and transmission [3,4,5,6,7]. Invasive neozoa have the capability to serve as additional host and vector species for various infectious pathogens, which might increase the possibility of disease spread [8]. 

Raccoon dogs (*Nyctereutes procyonoides*) and raccoons (*Procyon lotor*) are two of the most successful invasive alien species in Europe [9], originating in East Asia [10] and North America [11], respectively. Being omnivorous, they are discussed as predators of native fauna and might be competing for natural resources with native predators [12,13,14]. Both species have a high reproductive capacity and are able to adapt to different habitats [8,15]. They have expanded their geographic distribution and increased in abundance in Europe in the last decades [9]. Large-scale climatic habitat suitability for the two species is present in Europe and is likely to increase under upcoming climate change [16]. Additionally, these species have the potential to act as reservoirs for numerous infectious agents such as rabies lyssavirus (RABV), canine distemper virus (CDV), *Trichinella* spp., *Baylisascaris procyonis*, *Echinococcus multilocularis* and various vector-borne diseases, such as, for example, *Borreliella* spp. and *Rickettsia* spp. [5,8,15,17,18]. Their host and vector potential allows pathogens to increase in the environment and to extend their geographical range [5,9,15,19,20]. Therefore, raccoon dogs and raccoons have the capability to threaten biodiversity as well as human and animal health [9,14,21]. 

An increasing incidence and diversity of vector-borne infections and zoonoses with one-health relevance has been observed in recent years [3,22], which may also be influenced by improved technical diagnostics and epidemiological techniques [1].

Leptospirosis has emerged as a globally important infectious disease, being the most common bacterial zoonosis in humans worldwide [7,22]. The genus *Leptospira* consists of various species which are gram-negative spirochetes and can be divided into at least 300 serovars [23]. A broad range of infected wild and domestic vertebrates serve as reservoir hosts, which shed the bacteria via infected urine [22,24]. Humans can be infected either by direct contact with an infected animal or by indirect contact with contaminated material, e.g. soil or water [7,24]. Leptospirosis in humans may result in a life-threatening fever with kidney and/or liver failure, as well as severe pulmonary haemorrhage [22]. 

*Borreliella* spp. cause lyme borreliosis, a multisystemic inflammatory disorder and the most prevalent arthropod-borne disease in the northern hemisphere [25]. The genus *Borrelia* was initially described by Swellengrebel in 1907 [26], whereas Adeolu and Gupta dived the genus into two genera: *Borrelia* and *Borreliella* with species of the *B. burgdorferi* (s.l.) complex belonging to the *Borreliella* genus [26,27,28,29]. *Borreliella* spp. are spirochetes, which depend on a host and vector to maintain their life cycle [30]. In Europe, the tick species *Ixodes* (*I.*) *ricinus* is the primary vector, while small and medium-sized mammals and ground-feeding birds serve as sylvatic maintenance hosts and reservoirs of *Borreliella* spp. [1,31]. 

*Rickettsia* spp. are obligate intracellular bacteria, comprising pathogens of the spotted fever group (SFG) causing tick-borne rickettsiosis in humans [32,33]. Clinical signs include fever, rash, headache and myalgias, resulting in a mild to severe and potentially fatal disease [34]. Arthropods are involved in their infection cycle with various wild and domestic animals as hosts [35,36]. *Rickettsia* spp. are prevalent pathogens found in *I. ricinus* and *Dermacentor* (*D.*) *reticulatus* ticks in Europe [37].

Wild carnivores are often infested with *I. ricinus*, the most widespread and medically important European tick [38]. *I. ricinus* has a broad host range [39,40,41,42,43,44], including raccoon dogs [20,38,45] and raccoons [46], which among other vertebrate hosts makes them potential reservoir hosts. 

Literature on vector-borne pathogens in raccoon dogs and raccoons in Europe is scarce. So far, only a few studies have been carried out on animals from Germany [19,47], Austria [9], the Czech Republic [48,49], Spain [4,50] and Poland [6,38,47,51], investigating the presence of the tick-borne pathogens *Babesia* spp., *Anaplasma* spp., *Ehrlichia* spp., *Hepatozoon* spp., *Borrelia* spp., *Borreliella* spp., *Bartonella* spp. and *Neoehrlichia mikurensis*, respectively [5,49]. 

To the authors‘ knowledge, the presence of *Leptospira* spp., *Borreliella* spp. and *Rickettsia* spp. has not been investigated in raccoon dogs from Germany so far. In raccoons originating from the German federal states Mecklenburg-Vorpommern (Müritz National Park), Berlin, Lower Saxony and Baden-Württemberg, *Leptospira* spp. have been found [17,52,53]; nevertheless, the occurrence of *Borreliella* spp. and *Rickettsia* spp. has not been examined yet.

Hence, the aim of this study was to examine the presence of *Leptospira* spp., *Borreliella* spp. and *Rickettsia* spp. in raccoon dogs and raccoons from northern Germany, to investigate the ectoparasite burden, perform species identification of the pathogens and statistically analyse demographic factors and ectoparasite infestation potentially influencing the prevalence of the vector-borne pathogens.

## 2. Materials and Methods

### 2.1. Animals, Sample Preparation and Ectoparasite Burden

**Figure 1:**
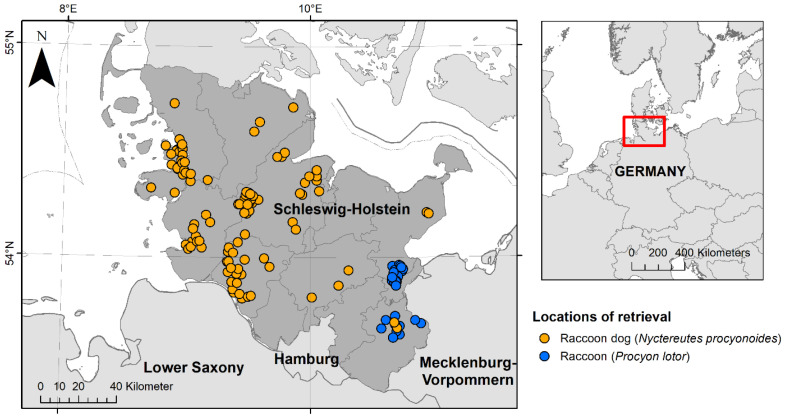
Locations of retrieval of 110 raccoon dogs (*Nyctereutes procyonoides*) and 30 raccoons (*Procyon lotor*) investigated during the health assessment study between 2021 and 2022 in the federal state of Schleswig-Holstein, Germany. Raccoon dogs are presented in orange and raccoons in blue.

During 2021 and 2022, 110 raccoon dogs and 30 raccoons that had been shot or found dead underwent post-mortem examinations at the Institute for Terrestrial and Aquatic Wildlife Research (ITAW) of the University of Veterinary Medicine Hannover as part of a health and risk assessment study of these invasive species in the federal state of Schleswig-Holstein, Germany (Figure 1) [54]. The animals were provided by hunters (A), who killed the animals as part of regular hunting practice in accordance with national hunting laws, or (B), who found them dead in their habitat. The killing of these animals did not require authorisation by an internal animal care and use committee or competent authority under Directive 2010/63/EU [55]. For all animals, the hunting area was documented, but no geocoordinates were collected. Of the raccoons, ~63% (19/30) were retrieved within the cities of Lübeck, Bad Schwartau and Ratekau. A modified necropsy protocol, according to Fähndrich et al. [56], was used. Selected biological data were collected as described by Klink et al. [54]. All animals were divided into one of two age classes (juvenile and adult) by size, teeth (deciduous or permanent) and tooth wear. During necropsy, all animals were examined macroscopically for ectoparasites, which were then collected and fixed in 70% ethanol. Ectoparasites were divided by taxonomic families and counted. All raccoons were frozen at −20 °C until further processing. Raccoon dogs were frozen at −70 °C for at least 96 h, to minimise the risk of *Echinococcus multilocularis* infections [57], and then stored at −20 °C. Four raccoon dogs were dissected right after arrival and two animals were only frozen at −20 °C due to logistical reasons; in these cases, personal protective equipment was used to reduce the infection risk. 

### 2.2. Tissue Preparation, DNA Extraction and Multilocus Sequence Typing

Kidney samples were taken individually; 0.6 g of sterile ceramic beads (sized 1.4 mm, Peqlab Biotechnologie, Erlangen, Germany) and 500 µL phosphate-buffered saline (PBS) were added. To the skin samples, 0.6 g of sterile steel beads (sized 2.8 mm, Peqlab Biotechnologie, Erlangen, Germany) and 500 µL PBS were added. All samples were homogenised in the Precellys^®^24 tissue homogeniser (Bertin Technologies, Montigny-le-Bretonneux, France), spleen samples at 5000 rpm for 2 × 30 s with 15 s breaks in between; skin samples underwent homogenisation twice at the same program settings. DNA extraction was performed on all samples with the QIAamp DNA Mini Kit (Qiagen, Hilden, Germany) according to the manufacturer’s recommendations for tissue DNA extraction. A spectrophotometer (NanoDrop^®^ 2000c, Peqlab Biotechnologie, Erlangen, Germany) was used to measure the quality and the quantity of the DNA samples.

### 2.3. PCR Methods

Kidney DNA samples were screened for the presence of *Leptospira* spp. and skin samples for *Rickettsia* spp. and *Borreliella* spp.:

Kidney DNA samples were tested by quantitative PCR (qPCR) for *Leptospira* spp., targeting the LipL32 gene (242 base pairs, bp), using the primers Lipl32-45F (5′-AAG CAT TAC CGC TTG TGG TG-3′) and LipL32-286R (5′-GAA CTC CCA TTT CAG CGA TT-3′) and probe LipL32 (5′6-FAM-AA AGC CAG GAC AAG CGC CG BHQ1-3′). The Qiagen QuantiTect Multiplex no Rox Kit (Qiagen, Hilden, Germany) was used. As a positive control, DNA from a laboratory strain of the *Leptospira kirschneri* serovar Grippotyphosa in a 1:10 dilution was used [23].

Skin DNA samples were tested by qPCR for *Rickettsia* spp., targeting the gltA gene (70 bp), using the primers Pan Rick gltA_2 for (5′-ATAGGACAACCGTTTATTT-3′) and Pan Rick gltA_2 rev (5′-CAAACATCATATGCAGAAA-3′) and the probe Pan Rick gltA_3 taq (5′-6FAM-CCTGATAATTCGTTAGATTTTACCG-TMR-3′). The Roche LightCycler FastStart DNA Master HybProbe Kit (Roche, Basel, Switzerland) was used. As a positive control, *Rickettsia helvetica* DNA from *Ixodes ricinus* ticks was used. 

Skin DNA samples were tested by qPCR for the presence of *Borreliella* spp., targeting the p41 gene (96 bp), using the primers FlaF1a (5′-AGCAAATTTAGGTGCTTTCAA) and FlaR1 (5′-GCAATCATTGCCATTGCAGA) and probe FlaProbe1 (5′-6 FAM-TGCTACAACCTCATCTGTCATTGTAGCATCTTTTATTTG—BBQ) following the protocol by Schwaiger et al. [58] using the Qiagen QuantiTect Multiplex no Rox Kit (Qiagen, Hilden, Germany). As positive controls, isolates of *Borreliella* (*B.*) *valaisiana* (2.0 × 10^5^ cells/µL) and *B. afzelii* (2.0 × 10^5^ cells/µL) in a 1:10 dilution were used. 

The Mx3000P Real-Time Cycler (Stratagene, Agilent Technologies Deutschland GmbH, Waldbronn, Germany) was used for all qPCR reactions, including the negative controls of the DNA extractions and the negative controls for each performed qPCR. 

Multilocus Sequence Typing (MLST) was performed on samples that tested positive for *Leptospira* spp. with ct-values <34 and on *Borreliella* spp.-positive samples if the ct-values were <35. MLST was carried out as described by Schmidt et al. [59] and Król et al. [60]. 

### 2.4. Histopathology

During post-mortem examination, selected tissue samples for the histological examination were taken and processed as described by Klink et al. [54]. The pathological findings will be described elsewhere. On kidney sections from animals where qPCR confirmed the presence of *Leptospira* spp. Warthin–Starry silver staining according to standard protocol was performed in addition to routine Haematoxylin and Eosin (HE) staining. 

### 2.5. Statistical Analysis

Confidence intervals (95% CI) for the prevalence of pathogens were determined by the modified Wald method using GraphPad Prism v.4 (Graph Pad Software, San Diego, CA, USA). Chi-square and Fisher’s tests were used to test the prevalence levels for significant independence. The significance threshold was set at *p* = 0.05. The ectoparasite infestation rate was compared between groups with the Mann–Whitney U test.

## 3. Results

### 3.1. Animals 

In total, 110 raccoon dogs were dissected. The proportion of female and male raccoon dogs was almost balanced (61 female and 49 male animals, respectively), while the age groups were clearly dominated by juvenile animals, at 80% (88/110). Overall, 30 raccoons were investigated, with an almost balanced sex proportion of 16 females and 14 males. Juvenile raccoons were the dominant age group, at 67%. For details, see Table 1.

In total, 70 out of 110 raccoon dogs (63.64%; 95% CI: 0.5432 to 0.7204) were infested with ectoparasites. Also, an ectoparasitosis was present in 7 out of 30 raccoons (23.33%; 95% CI: 0.1152 to 0.4120). 

The most frequent macroscopically detected ectoparasites in both neozoa species were ticks (Ixodida), with ~63% of the examined raccoon dogs and 20% of examined raccoons being affected. Fleas (Siphonaptera), lice (Phthiraptera) and louse flies (Hippoboscidae) were also detected. For details, see Table 2. 

**Figure 2:**
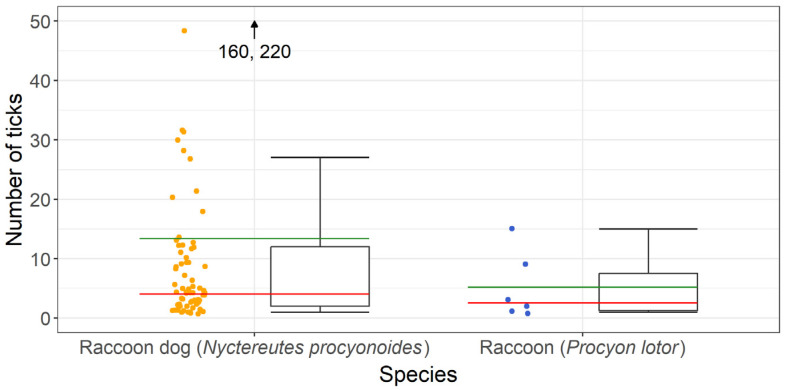
Number of ticks per individual collected from tick-infested raccoon dogs (*Nyctereutes procyonoides*) and raccoons (*Procyon lotor*); the mean intensity is presented in green and the median value in red.

In total, 925 ticks were collected from 69 raccoon dogs and 31 ticks were collected from 6 raccoons. A maximum of 220 ticks were found on one raccoon dog, while 15 ticks were the highest number found on a raccoon. A mean intensity of ~13 ticks per tick-infested raccoon dog and 5 ticks per raccoon infested with ticks was observed. Details are presented in Figure 2. 

Statistical analysis was performed, comparing the ectoparasite burdens of raccoon dogs and raccoons, which were not significantly different (*p* = 0.1310, t = 1.5192).

### 3.2. PCR and Statistical Analysis for Rickettsia spp., Borreliella spp. and Leptospira spp. 

As most animals were harvested by hunters, in some cases the carcasses were too destroyed to take all samples. Additionally, sample collection errors occurred. Therefore, there is a discrepant count between assessed animals and organs available for this study. 

In total, 136 skin samples were analysed for the presence of *Rickettsia* spp. and *Borreliella* spp. via performing qPCR. Furthermore, 138 kidney samples were investigated for *Leptospira* spp. 

*Rickettsia* spp. were not detected in both species. As shown in Table 3, two samples were positive for *Borreliella* spp., one obtained from a raccoon dog (1/107, ct-value: 37.52) and one from a raccoon (1/29, ct-value: 33.98). Of 138 kidney samples tested for *Leptospira* spp., 23 were positive. In total, in 21 out of 109 raccoon dogs (ct-values: 28.35–42.84) and in 2 out of 29 raccoons (ct-values: 34.95 and 36.59), *Leptospira* spp. were detected. The two raccoons were retrieved from the cities of Lübeck and Bad Schwartau. 

Statistical analysis of demographic data was performed on the occurrence of *Leptospira* spp. in raccoon dogs, showing no statistical difference between sexes (*p* = 0.8113) but statistical difference between age groups (*p* = 0.0066), with juvenile raccoon dogs being more often infected with *Leptospira* spp. than adults. Comparing the occurrence of *Leptospira* spp. in raccoon dogs and raccoons, raccoon dogs were more often infected than raccoons (*p* = 0.0450). 

The raccoon skin sample was tested by MLST and was positive for *B.afzelii.* Sequencing was not performed on the raccoon dog skin sample due to the exceeding ct-value (>35).

On six raccoon dog kidney samples, sequencing was performed, as the ct-values were below 34. MLST was performed, and in three cases *Leptospira* (*L.*) *borgpetersenii* and in one case each *L. kirschneri* and *L. interrogans* were identified. In one kidney sample, MLST was negative. 

### 3.3. Histopathology

**Figure 3:**
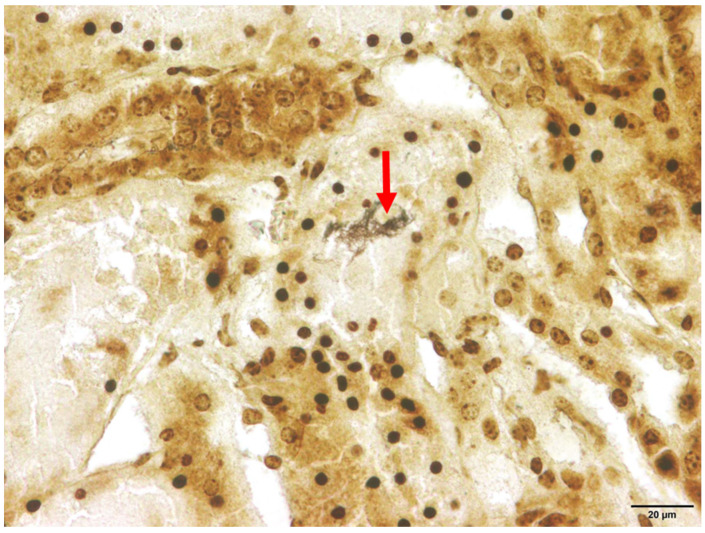
Renal histology of a *Leptospira* spp.-positive raccoon dog (*Nyctereutes procyonoides*). Within the cytoplasm of the renal tubular epithelium, numerous spiral-shaped bacteria corresponding to *Leptospira* spp. (marked with arrow) are present. Warthin–Starry silver stain.

Among the animals that tested positive for *Leptospira* spp., interstitial nephritis was found in two raccoon dogs (2/21) and both raccoons (2/2). Therefore, interstitial nephritis was present in 9.5% of the affected raccoon dogs and in all raccoons. In none of the investigated animals histologic lesions attributable to acute and severe leptospirosis were detected, which, apart from focal to diffuse interstitial nephritis, includes acute transient tubular injury or tubular epithelial necrosis [61]. Using Warthin–Starry silver staining, spiral-shaped bacteria, corresponding to *Leptospira* spp., were successfully demonstrated within the renal tubules in one animal (Figure 3).

## 4. Discussion

In this study, raccoon dogs and raccoons from Schleswig-Holstein were investigated for the presence of selected vector-borne and zoonotic pathogens, namely *Leptospira* spp., *Borreliella* spp. and *Rickettsia* spp., for the first time. In addition, analysis of demographic factors and ectoparasitic infestation in concordance with the pathogen prevalence was performed. 

Raccoon dogs and raccoons have the potential of being competent reservoirs of various pathogens, including rodent-borne and arthropod-borne pathogens [5,8,15,17,18]; however, there is a lack of data on the reservoir function of these neozoa in northern Germany. 

European raccoon dogs have not been examined for the occurrence of *Leptospira* spp. yet. Until now, *Leptospira* spp. were only identified in Korean raccoon dog faeces [62] and a brief report on leptospiral meningoencephalitis in a Japanese raccoon dog has been published [18]. The prevalence of *Leptospira* spp. in raccoon dogs in the present study was 19.44%, which is much higher than the prevalence of 6.7% reported in Korea [62]. In the Korean study, the leptospires were identified as *L. wolffii*, while in the raccoon dogs investigated in the present study, *L. borgpetersenii*, *L. kirschneri* and *L. interrogans* were identified, which are all pathogenic and can cause human leptospirosis, a zoonosis with worldwide distribution and global importance [22,63]. So far, in Germany, raccoons have been assessed for the presence of *Leptospira* spp. in the federal states of Mecklenburg-Vorpommern (Müritz National Park), Berlin, Lower Saxony and Baden-Württemberg [17,52,53]. The prevalence of *Leptospira* spp. in raccoons in the present study was 6.90%; in other studies, the prevalence was 20.6% in Berlin, 3.9% in Baden-Württemberg [17], 3.2% in Mecklenburg-Vorpommern [52] and 1.3% in Lower Saxony [53]. Therefore, the detected prevalence is highest in raccoons from an urban habitat, i.e., Berlin, followed by the examined raccoons in Schleswig-Holstein, which show a higher prevalence than in the other states. Of the investigated raccoons in this study, approximately 63% were retrieved within cities (Lübeck, Bad Schwartau and Ratekau), with the two positive animals being from Lübeck and Bad Schwartau. Geocoordinates were not taken as part of this study; therefore, the closeness to water bodies or human settlements of the other animals cannot be analysed. 

The closeness of urban raccoons to the dwellings of wild boars and rodents, especially rats, which are the main reservoir hosts for different pathogenic *Leptospira* spp., poses an increased chance for interspecies transmission in either direction [22,52]. Also, a prevalence of 18% of *Leptospira* spp. is described in wild boars in Berlin [64,65] and one case of human leptospirosis with wild boars as a possible source has been published [65]. Raccoon dogs are described to avoid human settlements [66], whereas raccoons reach high population densities close to human or agricultural settlements [67] and therefore might pose a higher risk of disease spread to humans. On the other hand, cohabitation of burrows of raccoon dogs with other carnivores is described [68], while to date interactions of raccoons with comparable predator species, e.g. the European badger (*Meles meles*) and the raccoon dog, have not been observed [67]. Thus, their life habits and the observed prevalence of 19.4% of *Leptospira* spp. in raccoon dogs might bear the potential of transmission of pathogens, especially to other carnivore species. Nevertheless, the presence of *Leptospira* spp. in both species bears the potential of transmission of zoonotic pathogens to domestic animals and humans, as well as other wildlife species, and both species should be considered as reservoir species for *Leptospira* spp. The reservoir function is further supported by the absence of attributable pathologic findings, e.g. focal or diffuse interstitial nephritis and acute transient tubular injury or tubular epithelial necrosis [61] in the kidneys of 90.5% of the infected raccoon dogs. Nevertheless, raccoon dogs are susceptible to disease, as in a raccoon dog from Japan leptospiral mengingoencephalitis was diagnosed with the histologic sections of the kidneys revealing diffuse lymphoplasmacytic interstitial nephritis [18]. Raccoons are also susceptible to disease and may not only serve as asymptomatic reservoirs, as interstitial nephritis was found histologically in raccoons originating from Germany, including in the present study and in the USA [52,69]. In the present study, the detected interstitial nephritis was overall rather mild and most likely subclinical in all cases.

Furthermore, the main food sources of raccoon dogs identified in Germany are small mammals, amphibians, birds, carrion, insects and plants [70,71], with small mammals making up 37.8% of consumed components [70]. In contrast, the most frequently detected food items for raccoons were invertebrates followed by plant-based food [72]. 

As raccoon dogs tend to feed more frequently on small mammals in comparison to raccoons, ingestion of infected prey should be considered as a possible transmission route, as prevalence of *Leptospira* spp. in raccoon dogs was significantly higher (19.44%) compared to 6.90% in raccoons. 

We performed statistical analyses on the occurrence of *Leptospira* spp. in the different age classes of raccoon dogs, and the *p* value showed a significantly higher occurrence in juveniles, indicating that juveniles might be more susceptible to the disease. Nevertheless, 80% of the assessed raccoon dogs were juveniles, which could also be a possible explanation for detecting *Leptospira* spp. only in this age class. 

Ectoparasitosis was a frequent finding in both species, with 64% of raccoon dogs and 23% of raccoons being affected. The most common ectoparasites were ticks, which were detected in approximately 63% of examined raccoon dogs and 20% of the examined raccoons. In a Polish study, 82.3% of the examined raccoon dogs were found to have ticks [38], while in a German study only 8.3% of the investigated raccoons were infested with ticks [52]. Nevertheless, the detection of ectoparasites might be biased by the sampling technique, as most ectoparasites dismount their host soon after its death [52]; for example, in a study conducted in the USA, prevalences of up to 92% for ticks were recorded in raccoons that were sampled while being anaesthetised [73]. The infection rates with different ectoparasite species might be even higher if examination takes place prior to transportation and freezing. Ticks are vectors for both *Borreliella* spp. and *Rickettsia* spp. [1,37]. There is a possible reservoir function of raccoon dogs for both, ticks and tick-borne diseases. The observed co-inhabitation of burrows with the red fox (*Vulpes vulpes*) and European badger may contribute to the maintenance of overlapping transmission cycles [38]. 

In Poland, different *Borreliella* spp. were identified in both raccoon dogs and raccoons [38,51]. In one study, *B. garinii* was found to be most frequent species detected in raccoon dogs, followed by *B. afzelii*; both pathogens are described as the dominant spirochetes found in ticks in Europe [38]. In the second study, *B. afzelii* was the only species found in both, raccoon dogs and raccoons, with prevalences of 2.0% and 23.5%, respectively [51]. In our study, one raccoon dog and one raccoon were positive for *Borreliella* spp., which could be identified as *B. afzelii* in the raccoon. *B. afzelii* is one of the causative agents of lyme borreliosis, with rodents being described as principal reservoirs [74,75]. Domestic dogs, badgers and raccoon dogs are also susceptible [38,76]. Even if the prevalence of *Borreliella* spp. in both species was low and the sample size of raccoons was small, both species should be considered as possible reservoirs for this pathogen in Schleswig-Holstein, Germany.

Neither of the species has been investigated for the presence of *Rickettsia* spp. in Europe before [5]. In the present study, *Rickettsia* spp. were not detected in raccoon dogs or raccoons. Nevertheless, serological studies on the occurrence of *Rickettsia* spp. in Japanese [77] and South Korean [78] raccoon dogs do exist, and one study using molecular detection was carried out in Korea [79]. So far, only *Rickettsia* spp.-specific antibodies have been confirmed in South Korean raccoon dogs, with prevalences of 30.5% and 41.6%, tested via an indirect fluorescent antibody test [78]. In two studies in Japan, molecular detection of *Rickettsia* spp. in raccoons was performed [80,81]. *Rickettsia* (*R.*) *helvetica* and *R felis* were among the identified species, with prevalences between 0.1 and 1.6% [80,81]. Both pathogens are also present in Europe and belong to the six *Rickettsia* species present in Germany [82,83,84]. Tufts et al. [85] confirmed *Rickettsia* spp. by PCR with a prevalence of 7.7% (3/39) in assessed raccoons originating from the USA. Previous studies confirmed the susceptibility of both neozoa species to *Rickettsia* spp., for raccoon dogs so far only in native (Korea and Japan) ranges and for raccoons in native (USA) and introduced (Japan) ranges [5]. *Rickettsiae* are transmitted by various types of arthropods, including ticks, fleas, mites and lice [86]. The major host for *R. helvetica* is *I. ricinus*, while *R. felis* is mainly transmitted by the cat flea (*Ctenocephalides felis*), but was also detected in *I. ricinus* in Germany [82,84]. A possible explanation for not detecting *R. felis* in the present study could be the observed low infestation rate with fleas. Even if *Rickettsia* spp. and suitable arthropod vectors are present in Germany, a possible explanation for the absence of this pathogen in raccoon dogs and raccoons could be that *I. ricinus* is not present in their autochthonous range [30] and therefore is a foreign ectoparasite to both species. Additionally, coevolutionary dynamics may play a role. A variety of host defences to parasitism exists in nature, including immune defences such as resistance and tolerance [87]. Therefore, raccoon dogs and raccoons as neozoa might not be part of the transmission cycle of *Rickettsia* spp. in Germany yet. 

A follow-up study of the presence and prevalence of *Rickettsia* spp. and *Borreliella* spp. in the collected ectoparasites is intended to further evaluate the role of raccoon dogs and raccoons as reservoirs for these tick-borne pathogens in the federal state of Schleswig-Holstein. 

In conclusion, the results of this study show that raccoon dogs and raccoons in Schleswig-Holstein should be considered as reservoirs for pathogenic *Leptospira* spp. Also, the present study confirms the presence of *Borreliella* spp. in both species. Further research is necessary to rule out the reservoir function of both species for tick-borne pathogens. Their high reproductive rate, ability to adapt to different habitats, omnivorous feeding behaviour and dispersal capacity result in growing populations, which pose risks of disease spread, interspecies transmission and disease persistence. Therefore, long-term health monitoring of both species, including infectious disease surveillance and expansion of the study area, is essential to evaluate their potential risk to human and animal health. 

## Figures and Tables

**Table 1:** Age classes of 110 investigated raccoon dogs (*Nyctereutes procyonoides*) and 30 raccoons (*Procyon lotor*).

Species	Juvenile	Adult	Total
Raccoon dog	88	22	110
Raccoon	20	10	30

**Table 2:** Ectoparasites detected in 110 raccoon dogs (*Nyctereutes procyonoides*) and 30 raccoons (*Procyon lotor*).

Parasites	Raccoon Dogs (*n* = 110)	Raccoons (*n* = 30)
Ixodida	69	6
Siphonaptera	5	1
Phthiraptera	5	1
Hippoboscidae	5	0

**Table 3:** Vector-borne pathogens detected in 110 investigated raccoon dogs (*Nyctereutes procyonoides*) and 30 raccoons (*Procyon lotor*).

Pathogen	Raccoon Dogs	Raccoons
*Rickettsia* spp.	0/107 (0% 95 CI 0)	0/29 (0% 95 CI 0)
*Borreliella* spp.	1/107 (0.93% 95 CI < 0.01–5.62)	1/29 (3.45% 95 CI < 0.01–18.63)
*Leptospira* spp.	21/109 (19.44% 95 CI 3.01–27.97)	2/29 (6.90% 95 CI 0.85–23.03)

## Data Availability

The data presented in this study are available in this published article and its Supplementary File S1.

## Supplementary Materials

The following supporting information can be downloaded at https://www.mdpi.com/article/10.3390/pathogens13030270/s1. Supplementary File S1.
